# Characterization of a Merkel Cell Polyomavirus-Positive Merkel Cell Carcinoma Cell Line CVG-1

**DOI:** 10.3389/fmicb.2018.00713

**Published:** 2018-04-11

**Authors:** Celestino Velásquez, Yutaka Amako, Alexis Harold, Tuna Toptan, Yuan Chang, Masahiro Shuda

**Affiliations:** ^1^Cancer Virology Program, University of Pittsburgh Medical Center Hillman Cancer Center, Pittsburgh, PA, United States; ^2^Department of Microbiology and Molecular Genetics, University of Pittsburgh, Pittsburgh, PA, United States; ^3^Department of Pathology, University of Pittsburgh, Pittsburgh, PA, United States

**Keywords:** Merkel cell polyomavirus, MCV, Merkel cell carcinoma, MCC, cell line

## Abstract

Merkel cell polyomavirus (MCV) plays a causal role in ∼80% of Merkel cell carcinomas (MCC). MCV is clonally integrated into the MCC tumor genome, which results in persistent expression of large T (LT) and small T (sT) antigen oncoproteins encoded by the early locus. In MCV-positive MCC tumors, LT is truncated by premature stop codons or deletions that lead to loss of the C-terminal origin binding (OBD) and helicase domains important for replication. The N-terminal Rb binding domain remains intact. MCV-positive cell lines derived from MCC explants have been valuable tools to study the molecular mechanism of MCV-induced Merkel cell carcinogenesis. Although all cell lines have integrated MCV and express truncated LT antigens, the molecular sizes of the LT proteins differ between cell lines. The copy number of integrated viral genome also varies across cell lines, leading to significantly different levels of viral protein expression. Nevertheless, these cell lines share phenotypic similarities in cell morphology, growth characteristics, and neuroendocrine marker expression. Several low-passage MCV-positive MCC cell lines have been established since the identification of MCV. We describe a new MCV-positive MCV cell line, CVG-1, with features distinct from previously reported cell lines. CVG-1 tumor cells grow in more discohesive clusters in loose round cell suspension, and individual cells show dramatic size heterogeneity. It is the first cell line to encode an MCV sT polymorphism resulting in a unique leucine (L) to proline (P) substitution mutation at amino acid 144. CVG-1 possesses a LT truncation pattern near identical to that of MKL-1 cells differing by the last two C-terminal amino acids and also shows an LT protein expression level similar to MKL-1. Viral T antigen knockdown reveals that, like other MCV-positive MCC cell lines, CVG-1 requires T antigen expression for cell proliferation.

## Introduction

Merkel cell carcinoma (MCC) is an aggressive skin cancer associated with immunosuppression and ultraviolet exposure (Lemos and Nghiem, 2007; Heath et al., 2008). The incidence of MCC is approximately 1,500 cases per year in the United States, which has increased threefold over the past two decades. The discovery of Merkel cell polyomavirus (MCV) in 2008 from MCC lead to a more precise understanding of the pathogenesis of these tumors (Feng et al., 2008).

Merkel cell polyomavirus was discovered by digital transcriptome sequencing of MCC and was found to be clonally integrated in 70–80% of these tumors. While the viral integration sites in the tumor genome are random (Feng et al., 2008; Sastre-Garau et al., 2009; Martel-Jantin et al., 2012), MCC tumor cells express the viral T antigen early gene. The MCV early locus is essential for virus replication and encodes four viral proteins: (1) large T (LT), (2) small T (sT), and (3) 57kT, which are expressed by alternative splicing mechanisms (Shuda et al., 2008), and (4) alternate frame of the large T open reading frame (ALTO) produced through alternative translation initiation (Carter et al., 2013). The MCV LT protein directly replicates the viral genome through its carboxyl (C)-terminal DNA origin binding domain (OBD) and DNA helicase activities, while sT enhances viral DNA replication by stabilizing LT (Kwun et al., 2009, 2013). The functions of 57kT and ALTO in the viral lifecycle have not been determined. Viral sequence analyses in MCC consistently show that tumor-derived LT antigens contain mutations that prematurely truncate the C-terminal helicase domain, while preserving the N-terminal tumor suppressor targeting domains for Rb-binding and DnaJ chaperone-binding functions (Grundhoff and Fischer, 2015; Wendzicki et al., 2015; DeCaprio, 2017). Most MCC tissues, if not all, express the truncated LT and intact sT proteins, whereas 57kT and ALTO have never been detected in tumor tissues (Shuda et al., 2009; Rodig et al., 2012).

These viral features in MCC tissues – clonal integration, LT helicase ablation, and T antigen expression - are also observed in the MCV-positive MCC cell lines. Since the first description of the MCV-positive MKL-1 cell line (Rosen et al., 1987; Shuda et al., 2008), 16 additional cell lines, including MKL-2, WaGa, BroLi, LoKe (Houben et al., 2010, 2012), MCCL-3, MCCL-11, MCCL-12 (Fischer et al., 2010), PeTa, AIDo, WoWe, HeRo, KaRi (Houben et al., 2013), MS-1 (Guastafierro et al., 2013), UM-MCC13, UM-MCC29, and UM-MCC565 (Verhaegen et al., 2014) have been established. The complete viral sequence has been only determined in nine MCC cell lines. The use of MCV-positive cell lines in T antigen knockdown studies revealed that T antigen expression is essential for cell proliferation in most MCV-positive MCC cell lines, consistent with the oncogenic role of T antigens in Merkel cell carcinogenesis (Houben et al., 2010).

Both MCC and small-cell lung carcinoma (SCLC) are neuroendocrine tumors, and MCC cell lines share cytological and histochemical similarities (Carney et al., 1985; Leonard et al., 1993). According to SCLC cell line classification, MCC cell lines can be divided into two groups: (1) classic and (2) variant, as defined by their immunohistochemical expression of markers including neuron-specific enolase and chromogranin A. In addition, cell lines can be classified further into four subtypes (type I-IV) based on growth morphology, colony shape, and aggregation (Leonard et al., 1995). Interestingly, with rare exceptions (Fischer et al., 2010), most MCV-positive MCC cell lines identified to date conform to the classic type with high neuroendocrine marker expression and exhibit type III morphology in which cells grow in loosely aggregated clusters. Gene expression analyses in MCV-positive MCC cell lines demonstrated that classic virus-positive cell lines such as MKL-1 and WaGa mirror the transcriptome profile of MCC tumors (Daily et al., 2015).

Despite their similarities, MCV-positive MCC cell lines show variability in morphology, growth characteristics, and tumorigenicity. Mouse xenograft studies using four MCV-positive MCC cell lines have demonstrated significant differences in the latency required for grafted tumor cell lines to grow (Dresang et al., 2013). In the same study, Dresang et al. also showed that response to the survivin inhibitor YM155 varies between cell lines (Dresang et al., 2013). While individual cancer cell lines possess unique host genetic mutations, phenotypic differences seen in the cell lines may be dependent, in part, on viral factors including the copy number of integrated viral genome and viral T antigen expression levels (Houben et al., 2010; Guastafierro et al., 2013). Further, each MCC tumor and MCV-positive cell line contains unique LT truncation mutations that produce LT proteins of different sizes.

Here, we describe the characteristics of a novel early passage MCV-positive MCC cell line, CVG-1, derived from a metastatic MCC explant and that exhibits LT truncation pattern and integrated viral copy number similar to that of the previously established MKL-1. While we found a unique leucine (L) to proline (P) missense mutation in the CVG-1-derived sT, this substitution mutation did not affect the transformation activity of CVG-1 sT in rodent fibroblasts. In fact, CVG-1 cell proliferation shows oncogenic addiction to viral T antigen expression similar to other MCV-positive MCC cell lines. Despite these similarities in virological features, a striking difference in tumor cell morphology was seen in CVG-1. Thus, establishment of CVG-1 not only extends the repertoire of MCV-positive cell lines but also serves as a tool to investigate host cell factors that contribute to the variations in growth morphology and tumorigenicity observed in other MCC cell lines.

## Materials and Methods

### Tissues and Cell Lines

Metastatic MCC to the left cervical lymph nodes was surgically resected from a 70-year-old female with a previous history of MCC. After surgical resection, the tissue [Collaborative Human Tissue Network (CHTN)] was placed in the RPMI1640 medium. All specimens were tested under University of Pittsburgh Institutional Review Board-approved guidelines. Patient informed consent was obtained by the participating CHTN hospitals in accordance with the Declaration of Helsinki. Tumor tissue was minced to disaggregate tumor cells. To adapt cells to cell culture, cells were initially cultured in the RPMI medium containing 20% heat-inactivated fetal bovine serum (FBS), 0.01% penicillin/streptomycin, 0.01% fungizone, 0.01% insulin–transferrin–selenium, 50 μM bathocuproine disulfate, and 1 mM L-cysteine. After 9 days, cells were transferred into RPMI medium containing 10% FBS and non-essential amino acids (NEAA, Mediatech). CVG-1 cells of passage <10 were cryopreserved in 10% DMSO in FBS. The experimental analyses in this study were carried out using cells at low passage (<40). Other MCC cell lines (MKL-1, MKL-2, and MS-1) were cultured in the RPMI medium supplemented with 10% FBS and NEAA.

### Immunohistochemistry

Formalin-fixed paraffin-embedded tumor tissue was stained according to a previously published protocol (Shuda et al., 2009). Briefly, slides were deparaffinized in xylene and rehydrated through a series of ethanol solutions. Endogenous peroxidase activity was blocked with 3% hydrogen peroxide for 15 min. Epitope retrieval was performed using 1 mM EDTA buffer, pH 8.0, at 125°C for 3 min and 90°C for 15 s in an antigen retrieval chamber (Decloaking Chamber, Biocare Medical). Following blocking with serum-free Protein Block (Dako), MCV large T antigen was immunostained with mouse monoclonal antibody CM2B4 (1 μg/ml) diluted in antibody buffer (1% BSA, 0.1% gelatin, 0.5% Triton-X, 0.05% sodium azide in PBS, pH 7.4) for 1 h at room temperature. After extensive rinsing in 1X tris-buffered saline solution (TBS), sections were incubated with mouse Envision Polymer (Dako) for 30 min at room temperature, reacted with diaminobenzidine (Dako), and counterstained with hematoxylin (Dako).

### Immunoblotting

Cells were lysed in RIPA buffer (50 mM Tris–HCl, 150 mM NaCl, 0.1% SDS, 0.5% Triton X, 0.5% sodium deoxycholate, pH 7.0) containing proteinase inhibitor cocktail (Roche). Lysates were sonicated to shear genomic DNA, and protein concentration was determined by the colorimetric DC-Protein Assay kit (Bio-Rad). After protein lysates were mixed with Laemmli buffer and heat-denatured, 30 and 50 μg of total protein were resolved in 12% SDS-polyacrylamide gel for LT and sT detection, respectively. MCV LT protein was detected by CM2B4 (Shuda et al., 2009). MCV sT was detected by CM5E1 (Shuda et al., 2011; **Figure 2C**) or by CM8E6 (Kwun et al., 2009; **Figures 3B,C**).

### MCV Genomic Sequencing

Genomic DNA was extracted from CVG-1 cells using standard phenol-chloroform extraction methods. The MCV DNA in CVG-1 cells was then amplified using polymerase chain reaction (PCR) primer sets (Contig1 to Contig13 primers) as described previously (Feng et al., 2008). PCR reactions in this study were performed by Q5 Hot Start High-Fidelity DNA polymerase (NEB). Sanger sequencing was performed by MCLAB (San Francisco, CA, United States).

### Viral Copy Number Analysis and Quantitative Reverse Transcription (RT)-Polymerase Chain Reaction (PCR)

Subcellular fractionation was performed to isolate nuclei from MCC cells prior to genomic DNA extraction by Proteinase K/SDS buffer (10 mM Tris/HCl pH 8.0, 100 mM NaCl, 25 mM EDTA, 0.5% SDS, 0.1 mg/mL Proteinase K). After overnight incubation at 50°C, 100 ng/mL of RNaseA was added to the lysates and incubated for 1 h at 37°C. Extracted DNA was further purified by phenol-chloroform extraction and ethanol precipitation prior to quantitative PCR (qPCR). The obtained genomic DNA was dissolved in water and quantified by UV-Vis spectrophotometry. qPCR was performed to obtain absolute quantification using the aforementioned total T antigen primer set. In order to determine the absolute copy number, a dilution series of plasmid MCV-HF was used as a reference template DNA (1 ng MCV-HF plasmid is equivalent to 1.13 × 10^8^ molecules).

Total cellular RNA was extracted and purified from MCV-positive MCC cells using Purezol (Bio-Rad) per manufacturer’s instructions, treated with RNase-free DNaseI (NEB), and extracted again with Purezol reagent prior to cDNA synthesis. For cDNA synthesis, 500 ng of total RNA was subjected to first strand synthesis using the iScript cDNA synthesis kit (Bio-Rad). Subsequently, real-time PCR was performed using PowerUP SYBR Green Master mix to quantify total T antigen mRNA (sense: 5′-GCT CCT AAT TGT TAT GGC AAC AT-3′ and antisense: 5′-CAA CAT CCC TCT GAT GAA AGC-3′), sT mRNA (sense: 5′-TCC TTG GGA AGA ATA TGG AAC T-3′ and antisense: 5′-GCG AGA CAA CTT ACA GCT AAT AC-3′), and 18S rRNA (sense: 5′-GGA CAC GGA CAG GAT TGA CA-3′ and antisense: 5′-ACC CAC GGA ATC GAG AAA GA-3′). Real-time PCR reactions were triplicated for each sample and performed on the QuantiStudio 3 system (Applied Biosystems). The determined threshold cycle (Ct) values were used to calculate the mRNA abundance relative to CVG-1 using the delta-delta Ct method. The Ct values of 18S rRNA were used as reference.

### Transformation Assays

Rat-1 cell transformation assays were performed as described previously (Shuda et al., 2011). For doxycycline-inducible transgene expression, pLenti TRE.MCS EF.Puro-2A-rTet was modified from TLCV2 by removing the U6 promoter-stuffer fragment by *Kpn*I and *EcoR*I digestion, followed by displacing Cas9-2A-eGFP fragment with double strand DNA linker containing unique restriction sites using *Age*I and *Nhe*I restriction sites. TLCV2 was a gift from Adam Karpf (Addgene #87360). Then, codon-optimized wild-type MCV sT and the amino acid substitution mutants including L114P, LSD, as well as L142A were inserted into pLenti TRE.MCS EF.Puro-2A-rTet by unique *Age*I and *Sbf*I restriction sites. For lentivirus production, 293FT cells (Invitrogen) were transfected with pLenti TRE MCV sT, psPax2, and pMD2.G using Lipofectamine 2000 (Invitrogen). The medium was changed at 12 h after transfection, and the lentivirus produced in the culture supernatant was harvested at 72 h after transfection.

For the foci formation assay, Rat-1 cells were seeded in 6-well plate and infected with 150 μL of lentiviruses encoding multiple MCV sT variants. At day 3 post-infection, fresh growth medium with or without 0.5 μg/mL of doxycycline was added. Rat-1 cells were cultured for 11 days, and cell culture medium was refreshed every 3 days to maintain transgene expression. Cells were stained by 0.5% crystal violet to visualize transformation-associated foci. For soft agar colony formation assays, Rat-1 cells transduced with the aforementioned lentivirus constructs were treated with puromycin (3 μg/mL). After puromycin-resistant cells were selected, 1.0 mL (1.25 × 10^4^) of the cells containing 0.3% noble agar (Sigma) was overlaid on the 2 mL of growth medium containing 0.6% agar. Cells were cultured for 2 weeks to observe colony formation in the soft agar. Colony numbers were counted under a microscope. The data is presented as mean ± standard deviation (SD) (*N* = 3).

### shRNA Knockdown of the Viral T Antigen and Cell Proliferation Assays

A modified version of the enhanced 7SK Pol III promoter (e7SK) was used as described previously (Haraguchi et al., 2016). In order to express short-hairpin (sh) RNA under the strong e7SK promoter, we synthesized a DNA fragment of the e7SK promoter (gBlock, IDT) and inserted it into the pENTR1A vector (Addgene plasmid #17398) to generate the pENTR e7SK-Pro construct using *Dra*I and *EcoR*V restriction sites. Oligonucleotides used to generate previously described shpanT (5′-CCG GAA GAG AGG CTC TCT GCA AGC TCT CGA GAG CTT GCA GAG AGC CTC TCT TTT TTG-3′ and 5′-AAT TCA AAA AAG AGA GGC TCT CTG CAA GCT CTC GAG AGC TTG CAG AGA GCC TCT CTT-3′) or control shRNA (5′-CCG GCC TAA GGT TAA GTC GCC CTC GCT CGA GCG AGG GCG ACT TAA CCT TAG GTT TTT G-3′ and 5′-AAT TCA AAA ACC TAA GGT TAA GTC GCC CTC GCT CGA GCG AGG GCG ACT TAA CCT TAG G-3′) (Houben et al., 2010) were annealed and inserted into pENTR e7SK-Pro using *Age*I and *EcoR*I restriction sites to generate pENTR1A-e7SK-shpanT and pENTR1A-e7SK-shCtrl, respectively. These entry vectors were inserted into the pLenti Dest-puro lentiviral vector using LR-clonase II (Invitrogen) to produce pLenti e7SK-shpanT-puro and pLenti e7SK-shCtrl-puro. pLenti Dest-puro was constructed from pMuLE Lenti Dest-Neo (Addgene plasmid #62178) by replacing the neomycin phosphotransferase gene with the puromycin N-acetyltransferase gene containing *Sbf*I and *Xho*I restriction sites. Lentivirus production was performed as described in transformation assays section. pENTR1A no ccdB (w48-1) and pLenti Dest Neo were a gift from Eric Campeau and Paul Kaufman and from Ian Frew, respectively.

For cell proliferation assays, CVG-1 cells were transduced with e7SK-shpanT-puro and pLenti e7SK-shCtrl-puro lentiviruses in the presence of 1 μg/mL polybrene for 2 days and selected for 4 days by puromycin treatment (1 μg/mL) as previously described (Houben et al., 2010). At day 6 post-transduction, 2.5 × 0^4^ cells were seeded in a 96-well plate (day 0). Cell proliferation was measured using WST-8 (Wako) at days 1, 3, 5, 7, 9, and 11. OD values were normalized by values from day 1. The WST-8 formazan product was measured at 440 nm with a reference filter at 600 nm using a Synergy 2^TM^ Multi-Mode Microplate Reader (BioTek).

## Results

### Establishment of the MCV-Positive MCC CVG-1 Cell Line

**FIGURE 1:**
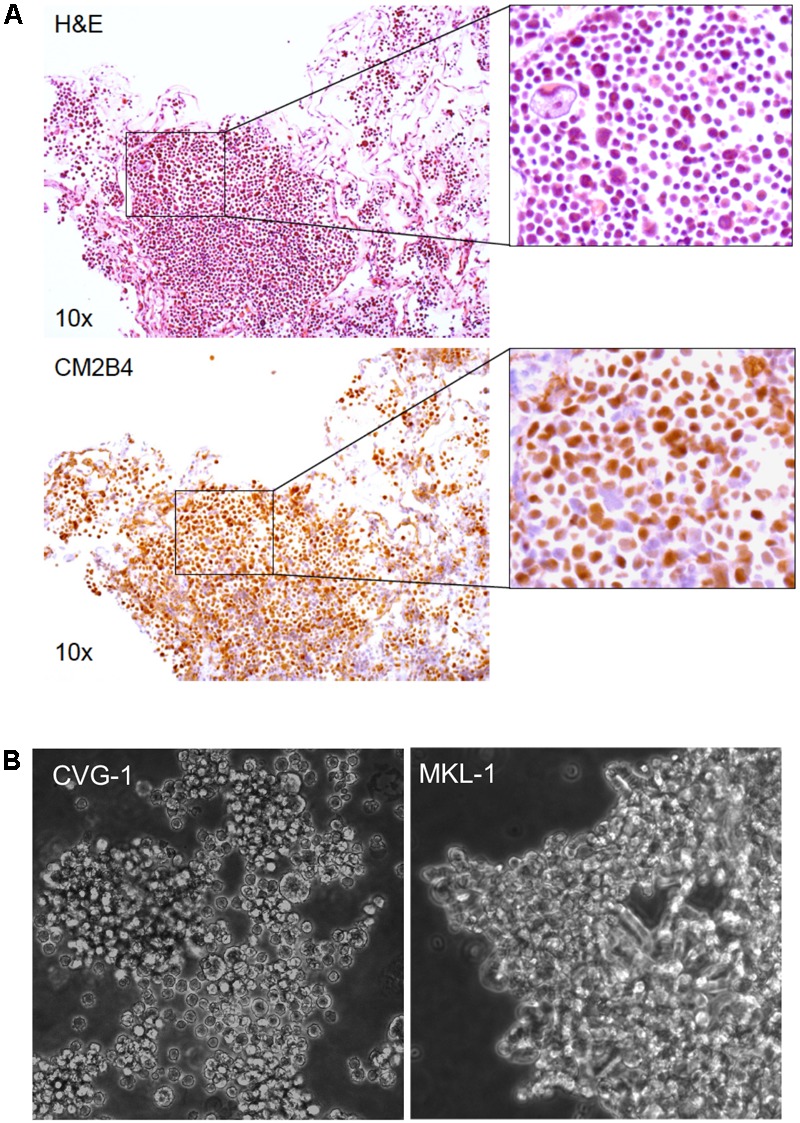
Establishment of the Merkel cell polyomavirus (MCV)-positive CVG-1 cell line from metastatic Merkel cell carcinomas (MCC). **(A)** Hematoxylin/eosin (H&E) staining (top) and MCV large T (LT) immunohistochemistry (bottom) of the metastatic MCC tissue that the CVG-1 cell line was established from. MCV LT-positive MCC tumor cells (brown nuclei) demonstrate significant variation in cell size (inset). **(B)** Morphology of the CVG-1 cell line under phase contrast microscopy. CVG-1 exhibits cell size variation and a small round appearance resembling lymphoid cell lines, as opposed to other MCV-positive MCC cell lines, such as MKL-1, which usually grow in loosely aggregated clusters.

A small piece of the metastatic MCC tissue used to establish MCC cell line CVG-1, was formalin-fixed and embedded in paraffin for hematoxylin/eosin (H&E) staining and immunohistochemistry. MCC tumor cell generally exhibit cytological features of monomorphic small round cells with high nuclear to cytoplasmic ratio. However, H&E staining of CVG-1 showed a heterogeneous cell population accentuated by large tumor cells (**Figure 1A**, Top panel). Immunohistochemistry using the MCV LT antibody (CM2B4) showed strong nuclear staining in both large and small tumor cells (**Figure 1A**, Bottom panel) confirming the presence of virus in each tumor cell.

Similar to the morphologic features of the parental MCC tumor, the CVG-1 cell line also displayed tumor cell size variation, having a mixture of large and small round cells (**Figure 1B**). We attempted to single-cell subclone large and small CVG-1 cells to demonstrate that both cells are LT-positive MCC but were unsuccessful. Nevertheless, since MCC tumor tissues are uniformly positive for LT protein, both large and small cells are likely to be MCV infected. CVG-1 cells grow in loose clusters in culture, whereas most MCC cells including MKL-1, MKL-2, and MS-1 grow in large, tightly cohesive aggregates (Guastafierro et al., 2013; **Figure 1B**). CVG-1 cells make contact with each other, but their contact involves only a small segment of the periphery of each cell.

### MCV Copy Number and Viral T Antigen Gene Expression in CVG-1

**FIGURE 2:**
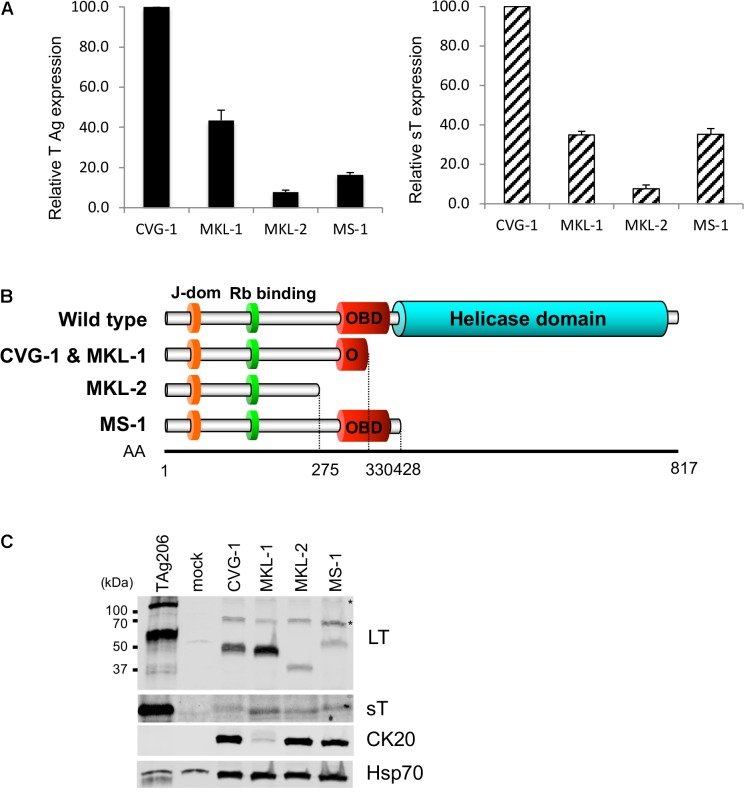
Expression of truncated MCV LT and small T (sT) antigens in CVG-1. **(A)** qRT-PCR analysis of T antigen transcripts in CVG-1 and three previously described MCV-positive MCC cell lines. The levels of total T antigen transcripts and sT transcript expressed in MCC cell lines were analyzed by the delta-delta Ct method to compare abundance of total T antigen (Left) and small T antigen transcripts (Right) relative to CVG-1 cells. Ct values from 18S rRNA amplification were utilized as an internal control. **(B)** Schematic representation of the LT truncation mutation in CVG-1, showing the ablation of the entire helicase domain. **(C)** Immunoblotting analysis of MCV LT and sT protein expression. MCV LT and sT protein expression was detected by CM2B4 and CM5E1 antibodies, respectively. CM2B4 detects full length LT (∼110 kDa) and multiply spliced 57kT (∼60 kDa) proteins in a positive control prepared from 293FT cells transfected with genomic MCV T antigen expression vector (TAg206), but not in a mock-transfected negative control. Cytokeratin 20 (CK20) expression was also detected as a marker of MCC. Hsp70 was used as a loading control. Asterisks indicate non-specific reactivities of CM2B4 antibody.

To determine the sequence of the MCV genome in CVG-1 cells, we amplified the viral DNA from CVG-1 using 13 pairs of contiguous primer sets that span the entire region of the MCV genome (Feng et al., 2008). The expected DNA fragments were successfully amplified with all primer sets used, indicating that the whole viral genome is present in CVG-1 cells. Next, the viral genome copy number was determined by qPCR using the previously characterized MKL-1, MKL-2, and MS-1 cell lines for comparison. CVG-1 contains the highest copy number of integrated MCV genome similar to MKL-1 (approximately 7 copies per cell), whereas MS-1 and MKL-2 contained relatively low copy numbers of MCV genome (4 copies and 2 copies per cell, respectively) (**Table 1**). Although genome copy numbers are similar between CVG-1 and MKL-1, analyses using qRT-PCR demonstrated that total T antigen mRNA and sT mRNA expression levels approximately 2.5-fold higher in CVG-1 than in MKL-1 (**Figure 2A**). MKL-2 expressed the lowest levels of T antigen mRNAs, consistent with previous results (Guastafierro et al., 2013; **Figure 2A**).

**Table 1:** MCV copy numbers in MCV-positive MCC cell lines.

Cell line	Absolute copy number/ng nuclear DNA	Copy number per cell (*N*)^∗^
CVG-1	1007.6 ± 30.1	3.63 ± 0.11
MKL-1	1014.6 ± 72.6	3.65 ± 0.26
MKL-2	280.2 ± 17.0	1.01 ± 0.06
MS-1	553.6 ± 18.3	1.99 ± 0.07

*^∗^Merkel cell polyomavirus (MCV) copy number per genome was calculated based on the assumption that 100 ng of nuclear DNA from merkel cell carcinomas (MCC) cells contains 27,777 copies of single human genome (3.6 pg per haploid cell).*

### Viral LT Truncation Mutation in CVG-1

Merkel cell carcinoma tissue-derived MCV harbors mutations that prematurely truncate the DNA helicase domain of LT (Shuda et al., 2008). These truncation mutations have also been identified in MCV-positive MCC cell lines (Guastafierro et al., 2013). Thus, we sought to map the LT truncation mutations in CVG-1. MCV DNA was amplified by the contiguous primers from genomic DNA extracted from CVG-1 cells, and the PCR products were subjected to direct sequencing (GenBank accession no. MH136801) (Feng et al., 2008). CVG-1 LT terminated at nucleotide position 1617 as a result of an A–T transversion mutation, generating a premature termination codon. In the DNA sequencing, while the CVG-1 cell contains ∼3.6 copies of integrated viral genomes per haploid cell (**Table 1**), there was no presence of multiple peaks/mixed signals, suggesting that all the copies of integrated MCV possess the identical truncation mutation. On the other hand, MKL-1 LT terminates at nucleotide position 1618 due to a 46-bp deletion that causes a frameshift. This frameshift mutation results in the addition of two unrelated amino acids prior to the termination codon. As their coding regions suggest, both CVG-1 and MKL-1 LTs are composed of 330 amino acids (**Figure 2B**), and the only difference between these was found to be two amino acids at their C-terminal ends—Ser-Asn for CVG-1 and Lys-Leu for MKL-1. As observed in the MKL-1 LT, the premature truncation mutation found in the CVG-1 LT resulted in the complete deletion of the OBD and helicase domains without the affecting Rb-binding domain (**Figure 2B**). The CVG-1 and MKL-1 cell lines are the first pair of cell lines that express the same length LT protein out of the nine MCV-positive MCC cell lines characterized to date. Next, we determined that the LT genetic truncation in CVG-1 has the same migration pattern as MKL-1 in SDS–PAGE (**Figure 2C**). Consistent with the LT truncation pattern observed in the MKL-1 cell line, the CVG-1 LT migrated at 50 kDa. The mobility of CVG-1 LT, however, was marginally slower than that of MKL-1. Furthermore, LT levels in CVG-1 were slightly lower than those in MKL-1, whereas the T antigen mRNA levels in MKL-1 were higher than those in MKL-1 cells (**Figure 2A**).

CVG-1 contains a unique missense mutation in the sT coding sequence (nucleotide position 536) that generates a Leu(L)-to-Pro(P) substitution at the amino acid position 114 of the sT protein. In the nine MCV-positive MCC cell lines in which the viral sequence has been defined, only one amino acid substitution has been found in three cell lines (MKL-2, PeTa, and WoWe), all at position 20 of the sT protein (Ala (A) to Ser(S)). This position corresponds to a common exon1 of the T antigen gene, and thus, both LT and sT proteins are affected by the A20S substitution. Thus, L114P is a unique MCV sT amino acid substitution found in CVG-1. In contrast to LT, sT protein expression is relatively comparable across all the cell lines tested, irrespective of differences in mRNA expression (**Figure 2C**). These results suggest that LT and sT protein expression may be regulated at the post-transcriptional level.

While no precise correlation was observed between T antigen mRNA and protein expression, MKL-1 and CVG-1 cells harboring higher copy numbers of the integrated MCV genome (**Table 1**) tend to express higher LT protein than MKL-2 and MS-1 cell lines, which contain lower copy numbers of viral genome (**Figure 2C**). We also confirmed protein expression of MCC marker CK20 by immunoblot. Consistent with previous results (Houben et al., 2010), all cell lines including CVG-1 express similar levels of CK20 except for MKL-1 which expresses significantly lower levels of CK20.

### Transforming Activity of CVG-1 sT With L114P Missense Mutation

**FIGURE 3:**
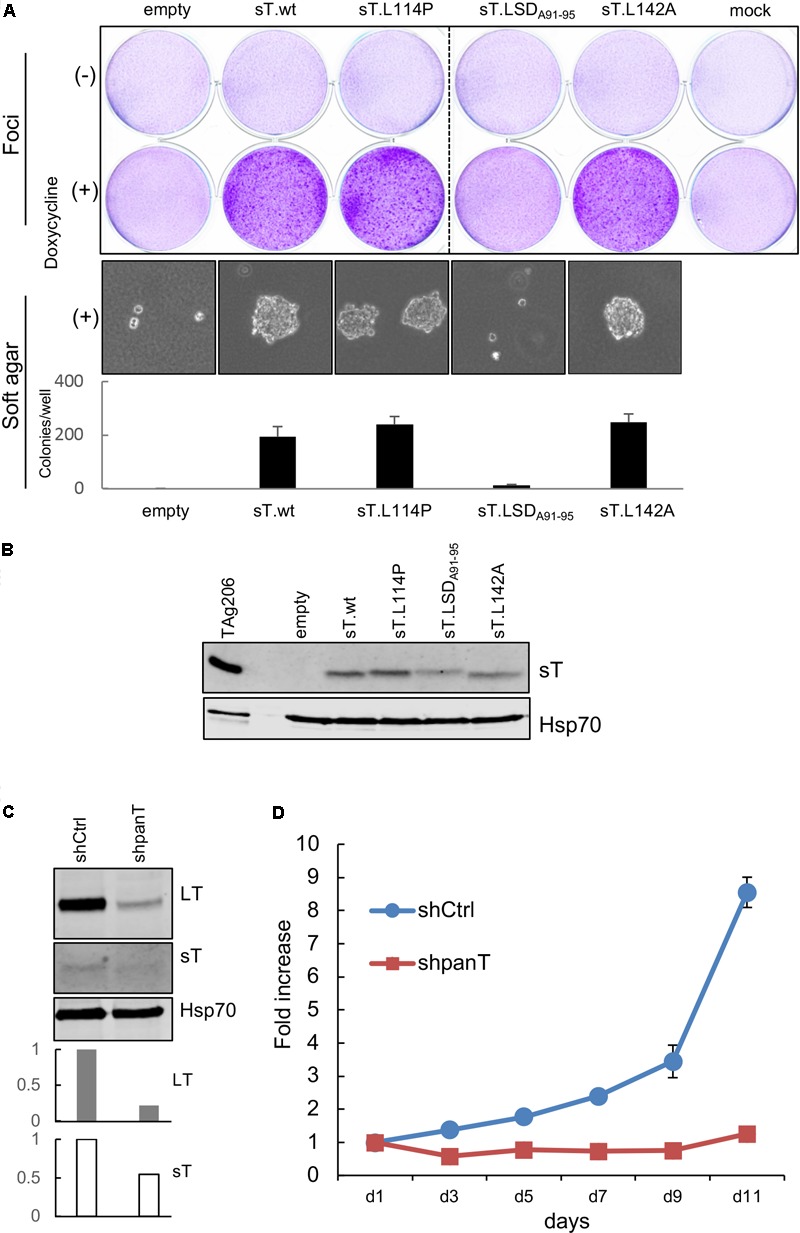
CVG-1 encodes transforming MCV sT and requires viral T antigen expression for cell proliferation. **(A)** Transformation activities of MCV sT L114P substitution mutant expressed in CVG-1. Foci-formation assay was performed in Rat-1 cells transduced with doxycycline-inducible lentiviral vectors that encode MCV sT and various substitution mutants in the presence or absence of 0.5 μg/mL doxycycline (Top). Soft agar colony formation assay was also performed in the presence of 0.5 μg/mL doxycycline (Bottom). The graph shows the average ± standard deviation (SD) of the number of soft agar colonies counted in triplicate wells. **(B)** Immunoblotting analysis confirming MCV sT protein expression in Rat-1 cells. A positive control lysates used in **Figure 2B** was loaded in the first lane. sT was detected by the CM8E6 antibody. Hsp70 was used as a loading control. **(C)** CVG-1 requires MCV T antigen expression for cell proliferation. CVG-1 cells were transduced with shRNA lentiviral vector targeting both LT and sT (shpanT) and control shRNA (shCtrl). After transduced cells were selected by puromycin, knockdown activity was confirmed by immunoblotting for LT, sT, and Hsp70 proteins. Graphs demonstrate knockdown activities of sT relative to shCtrl quantified for LT and sT proteins by LI-COR immunoblotting. T antigen protein expression was normalized by the Hsp70 protein expression. **(D)** CVG-1 cell proliferation requires viral T antigen expression. After knockdown was confirmed, cells were seeded in a 96-well plate, and the proliferation was assessed using the WST-8 assay.

Merkel cell polyomavirus sT is the major MCV oncoprotein that causes loss of contact inhibition and confers anchorage-independent growth capabilities in immortalized rodent fibroblasts (Shuda et al., 2011). This viral oncoprotein is a well-conserved protein across different isolates. In CVG-1 cells, we found a unique mutation that gives rise to a L114P amino acid substitution in the sT specific region. To date, this is the second substitution mutation identified in the sT protein in MCV-positive MCC cell lines. In a previous study, we found that the large T stabilization domain (LSD), a domain critical for the transformation activity of sT, is localized to amino acids 91–95 of the sT protein and is part of a disordered loop structure based on the predicted MCV sT structure (Kwun et al., 2013, 2015). Because the L114P mutation is in close proximity to LSD and proline is an amino acid that introduces sharp kinks in the polypeptide backbone, we wondered whether this newly identified sT mutation affects CVG-1 sT transformation activity. We used a previously described rodent cell transformation assay (Shuda et al., 2011). Rat-1 cells were transduced with lentiviruses expressing wild type sT and various sT mutants in the presence of doxycycline (500 ng/mL). Induction of L114P sT mutant expression in cells resulted in dense foci formation similar to that observed in cells expressing wild-type MCV sT and L142A PP2A-binding mutant, whereas cells expressing the non-transforming LSD mutant LSD_A91-95_ did not form dense foci (**Figure 3A**, Top). In parallel with the foci formation assay, we performed a soft agar colony formation assay and observed anchorage-independent growth in cells expressing the L114P sT mutant (**Figure 3A**, Bottom). Colony size and numbers were comparable between L114P and the other transforming MCV sT variants tested, suggesting that the L114P mutation does not affect the transforming activity of MCV sT. Comparable sT protein expression in lentivirus-transduced cells was confirmed by immunoblotting (**Figure 3B**).

### CVG-1 Requires Viral T Antigen for Cell Proliferation

While most MCV-positive MCC cell lines established to date require viral T antigen oncogene expression for proliferation, T antigen expression is dispensable only in one MCV-positive MCC cell line, LoKe (Houben et al., 2012). We examined whether CVG-1 also requires T antigen expression by using shRNA panT1 (shpanT) targeting common T antigen exon1 and control shRNA (shCtrl) (Shuda et al., 2011). After shRNA-transduced CVG-1 cells were selected by puromycin, knockdown was examined by quantitative LI-COR immunoblotting. Expression of LT and sT proteins in CVG-1 cells was reduced by 79% and by 46%, respectively, by knockdown with shpanT, as compared to cells transduced with shCtrl (**Figure 3C**). In addition, cell proliferation assays performed with WST-8 showed that T antigen knockdown by shpanT completely ablated the cell proliferation activity of CVG-1 cells (**Figure 3D**). Thus, similar to most MCV-positive cell lines, tumorigenicity of CVG-1 also depends on the viral T antigen expression.

## Discussion

Here we report the establishment of a new, early passage MCV-positive MCC cell line CVG-1. As seen in other MCV-positive MCC cell lines MKL-1(Shuda et al., 2008) and MCCL12 (Fischer et al., 2010), CVG-1 harbors multiple copies of the integrated MCV genome with a uniform LT truncation mutation, which may have resulted from the multiplication of a mutated single genome by rolling circle DNA replication (Starrett et al., 2017). Unlike most other MCV-positive MCC cell lines that grow in clusters, CVG-1 cells remain more disaggregated, and trypsin digestion was not required to isolate single cells. The CVG-1 cell line uniquely shows remarkable cell-size variation including large cells, even though MCC is defined as a small cell neoplasia. While we still do not know the specific cause of the size variation in CVG-1 cells, many type of cancers display morphological, proliferative, and functional heterogeneity, which arise from genetic mutations or cancer cell differentiation seen in cancer initiating cells or cancer stem cells. The former could be caused by the MCV sT antigen, which induces aneuploidy by compromising cell cycle checkpoint functions (Kwun et al., 2017). SCLC, another neuroendocrine cancer composed of cells capable of differentiating into neuronal and endocrine lineages, displays comparable morphologic heterogeneity and a gene expression signature similar to MCC (Salcido et al., 2010; Codony-Servat et al., 2016). Thus, self-renewal and cancer cell differentiation may also contribute to MCC tumorigenicity and give rise to morphological heterogeneity.

While the morphology of CVG-1 was unique among previously reported MCV-positive MCC cell lines, CVG-1 tumorigenicity is still dependent on MCV T antigen expression as in other MCV-positive cell lines. Out of seven cell lines tested for T antigen knockdown, only the LoKe cell line does not require T antigen expression for growth (Houben et al., 2012). Characterization of LoKe demonstrated that tumor cells harbor an Rb gene deletion that compensates for the oncogenic function of MCV LT expression in MCC (Houben et al., 2012; Hesbacher et al., 2016). However, it has been shown that Rb gene deletions/mutations are rare in MCV-positive MCC (Gonzalez-Vela et al., 2017; Harms et al., 2017). Thus, it remains likely that most MCV-positive MCC cell lines including CVG-1 require MCV T antigen expression to maintain their tumorigenicity.

The cell of origin in MCC remains unknown. It has been thought to be the epidermal Merkel cells based on the similarities in ultrastructure and gene expression between normal Merkel cells and MCC (Tang and Toker, 1978; Green et al., 1984; Battifora and Silva, 1986). However, it has recently been proposed that the cellular origin of MCV-positive cells may not be the Merkel cells (Zur Hausen et al., 2013; Sauer et al., 2017; Sunshine et al., 2018). Liu et al. (2016) have shown that MCV can infect dermal fibroblasts *in vitro*, but infection of Merkel cells has never been confirmed. Merkel cells are epidermal cells localized in the basal layer of the epidermis. While Merkel cells originate in the epidermis (Van Keymeulen et al., 2009), cutaneous MCCs typically involve only the dermis, and MCCs that develop in the epidermis are rare (<10%) (Smith et al., 1993). Recently, Verhaegen et al. (2017) reported transgenic mice that develop MCC-like tumors by expressing MCV sT and a Merkel cell lineage factor Atoh1 under the keratin 5 (KRT5) promoter, the activity of which is restricted to the basal layer of the epidermis (Ramirez et al., 1994). Interestingly, the pathogenesis of MCC-like tumors in this mouse model was restricted to epidermal layers, consistent with MCC-*in situ* or Merkel cell hyperplasia (McFalls et al., 2017). These data suggest the posibility that most MCV-positive dermal MCCs may originate from non-Merkel cells while MCC-*in situ*, which is confined to the epidermis, may arise from Merkel cells (Ferringer et al., 2005).

Since an animal model that mimics dermal MCC carcinogenesis has not been developed, MCC cell lines are useful tools to study the cellular origin of MCC. It has been shown that SV40 T antigen and human papilloma virus E6/E7 oncoproteins can reversibly transform primary human hepatocytes and human pancreatic duct epithelial cells without affecting normal diploid status (Kobayashi et al., 2000; Inagawa et al., 2014). The MCV-positive MCCs usually contain fewer genetic mutations and sustain normal karyotypes when compared to virus negative MCCs (Harms et al., 2017). Thus, some MCC cell lines may preserve normal genetic components that allow tumor cells to redifferentiate into untransformed, post-mitotic state cells with inhibition of T antigen expression. While most MCV-positive MCC cell lines become arrested after T antigen knockdown, a portion of cells commit non-apoptotic cell death as seen in MKL-1 (Houben et al., 2010). In early-passage cell lines like CVG-1 and MS-1 cells, however, many cells remain viable after T antigen knockdown and are arrested in G0/G1 (unpublished observation). Further molecular and cellular analyses in these early passage cell lines may lead to the identification of host genetic or functional features that represent the cellular origin of MCC.

Studies using MCC cell lines have revealed critical oncogenic pathways regulated by sT and LT. A recent study demonstrated that MCV sT binds to L-Myc and the EP400 histone acetyltransferase complex to activate L-Myc-mediated gene expression in MCC cells critical for MCC cell proliferation (Cheng et al., 2017). MCV LT expression in MCC activates the genes downstream of the E2F transcription factor by inhibiting the function of Rb through its LxCxE Rb-binding domain (Hesbacher et al., 2016). MCV-positive MCC is a unique cancer that has a gene expression signature similar to neuroendocrine Merkel cells. Because MCV T antigens alone are not sufficient to transform normal human fibroblasts (Cheng et al., 2017), MCC-specific oncogenic factors that are amplified in MCC such as L-Myc, may also play important roles in MCV-induced MCC carcinogenesis (Paulson et al., 2009; Cheng et al., 2017). Thus, MCC cell lines are essential tools to study the interplay between viral T antigens and MCC-specific host cell factors.

## Conclusion

We established a new, early passage MCV-positive MCC cell line CVG-1 from a patient with metastatic MCC. CVG-1 displays different morphologic features from other MCV-positive MCC cell lines, but nevertheless requires MCV T antigen for cell proliferation. While CVG-1 sT antigen contains a unique missense mutation, the mutant sT demonstrated similar transformation activity to prototypic sT in rodent cells. CVG-1 shows similarities to MKL-1 in viral copy numbers and LT truncation patterns. Further analyses of CVG-1 and MKL-1 may lead to the identification of critical host factors beyond the viral T antigen that contribute to the variations observed in MCC cell lines.

## Author Contributions

CV, YC, and MS established the CVG-1 cell line. MS conceived and designed the experiments. YA performed the qPCR experiments. MS performed the sequencing experiments. YA, AH, and MS performed the immunoblot and cell transformation experiments. TT performed the immunohistochemistry. MS, YA, and YC analyzed the data. MS, YC, CV, and YA wrote the paper.

## Conflict of Interest Statement

The authors declare that the research was conducted in the absence of any commercial or financial relationships that could be construed as a potential conflict of interest. The reviewer BA and handling Editor declared their shared affiliation.
